# Real-time peri-operative microcalcification detection in superficial breast tissues

**DOI:** 10.1038/s41598-025-01629-4

**Published:** 2025-05-16

**Authors:** Stefan D. van der Stel, Lorenzo Niemitz, Simon Sørensen, Lianne Feenstra, Stefan Andersson-Engels, Henricus J. C. M. Sterenborg, Theo J. M. Ruers, Ray Burke

**Affiliations:** 1 Department of Surgery, Netherlands Cancer Institute-Antoni van Leeuwenhoek, Amsterdam, The Netherlands; 2 Group Nanobiophysics, Faculty TNW, Twente University, Enschede, The Netherlands; 3 Biophotonics@Tyndall, IPIC, Tyndall National Institute, Cork, Ireland; 4 Department of Physics, University College Cork, Cork, Ireland; 5 Department of Biomedical Engineering and Physics, Amsterdam University Medical Center, Amsterdam, The Netherlands

**Keywords:** Breast cancer, Surgical oncology, Biomedical engineering, Imaging and sensing, Preclinical research

## Abstract

Surgical management of ductal carcinoma in situ (DCIS), particularly in cases involving suspicious morphology and orientation of microcalcifications, remains a primary treatment option. However, the lack of real-time technical assistance in the form of an intraoperative surgical tool for detection of microcalcifications in the resection margins presents a significant challenge. In the context of breast conserving surgery, ex-vivo imaging of excised breast tissues slices from 12 patients was conducted. By employing a cross-polarized multispectral microcamera setup for tissue visualization an imaging depth of up to 2 mm was achieved. The microcamera provides the clinician with a clear color image with magnification allowing features down to 50 μm to be seen on the resection surface. Mammography images were used for accurate cross-correlation, enabling the identification of microcalcifications in the microcamera images. Detection efficacy of microcalcifications in microcamera images was notably influenced by both calcification clustering and distribution depth within the tissue. Calcifications within the 2 mm range were detected through their distinct optical manifestations in relation to the adjacent tissues. Four independent reviewers—two medical and two technical—achieved an average sensitivity of 77.8%, specificity of 80.0%, and overall accuracy of 79.0%. This study demonstrates the potential of an integrated microcamera and cross-polarized setup for non-invasive, real-time detection of microcalcifications in superficial breast tissues. By focusing on the superficial 2 mm, this approach shows promising results and offers substantial opportunities for future research and clinical applications.

## Introduction

Since the introduction of breast cancer screening programs, and the implementation of the Breast Imaging Reporting and Data System (BI-RADS), breast cancer is often detected at early or premalignant stages^1^. In case of suspicious tissue alternations in screening mammography, additional in-hospital diagnostic examinations are required^2^. For example, 22,070 women from the preventive breast cancer screening program in the Netherlands were referred to the hospital for additional examinations in 2018^3^. A common reason for referrals is the presence of suspicious microcalcifications on mammography, in many cases without a visible tumor. Moreover, of all referrals in 2018, 34.7% of patients were referred for additional examination solely due to presence of microcalcifications^3^.

Microcalcifications are deposits of calcium in the lobular system of the breast and can be a sign of a (pre) malignant lesion called ductal carcinoma in situ (DCIS)^4^. DCIS is considered as precursor lesion of an invasive carcinoma (IC) of the breast and is characterized by isolated proliferation of malignant cells in the ductolobular system of the breast^5^. DCIS accounts for 20–30% of all breast cancer diagnosis^6^, and a key characteristic is that microcalcifications are present in 60–90% of DCIS lesions^7,8^. Despite being a common finding in breast mammographies in preventive screening programs, not all calcifications are related to DCIS. Hence, radiologists differentiate between benign and malignant calcifications based on size, morphological appearances and distribution^9^. Malignant calcifications are small, mostly intraductal, display fine linear, branching and pleomorphic morphological characteristics and are distributed linearly or segmentally, whereas benign calcifications are larger in size and distributed diffusely within the lobuli^10^.

In current treatment guidelines of breast cancer, surgery remains the best option to ensure complete removal of all malignant tissue^11^. However, DCIS is usually presented as scattered islands of malignant cells and not as a solid mass, making it increasingly difficult to distinguish from the surrounding healthy tissue in the surgical setting, resulting in up to 30% tumor-positive resection margins after postoperative histopathological examination^12^. Residual tumor cells in the resection margins are a major prognostic factor for local tumor recurrence in breast conserving surgery (BCS) and are more prevalent after DCIS-indicated surgeries compared to IC of the breast^13^.

Currently, the gold standard for resection margin assessment is histopathological evaluation by a pathologist^14^. However, this procedure can take up to multiple days, delaying the final diagnosis. To overcome this gap, multiple optical imaging techniques are being explored for resection margin assessment and tumor tissue differentiation. In the past, diffuse reflectance spectroscopy (DRS)^15–17^, hyperspectral imaging (HSI)^18,19^ and Raman spectroscopy^20^ showed promising results for characterizing tumor and healthy breast tissue. Despite showing the great potential in identifying solid tumors, characterizing smaller islands of DCIS with the aforementioned techniques remains challenging^16^. In recent years, the biology and physiology of microcalcifications have become a popular research topic, leading to a better understanding of their origin and involvement in breast cancer^4,20,21^. So far, however, little research has been reported into intraoperative visualization of microcalcifications. While various imaging techniques have been developed for preoperative detection and analysis of microcalcifications, their direct application during surgery remains limited. A review by Kennedy et al. discusses imaging approaches for intraoperative margin detection in breast-conserving surgery but does not specifically address real-time visualization of microcalcifications during the procedure^22^. Similarly, a review by Brahimetaj et al. highlights the use of micro-CT for detailed visualization of breast tissue and microcalcifications but focuses on ex vivo analyses rather than intraoperative applications^23^. These studies suggest that while imaging technologies have advanced in detecting and analyzing microcalcifications pre- and postoperatively, their real-time intraoperative visualization has not been extensively researched.”

Microcameras have become increasingly popular tools in the surgical workflow, facilitating real-time visualization through minimally invasive access points^24,25^. In particular, a microcamera can offer clinicians enhanced visualization of superficial tissues, by providing magnified perspectives of the surgical field. In BCS, the integration of microcameras holds particular promise by offering real-time assessment of the resection plane for presence of tumor or microcalcifications in case of DCIS. The main advantage here is that assessment of resection planes intraoperatively, provides the opportunity to excise additional tissue in the same surgical setting if indicated.

Recently, we presented a new cross-polarized microcamera setup, utilizing diffuse reflectance imaging at multiple wavelengths to visualize the superficial 2 mm, magnified up to 50 μm, within an experimental setup^26^. By offering enhanced visualization of the superficial 2 mm of the resection plane, this technique holds promise to aid surgeons in achieving accurate resection planes, while preserving as much healthy tissue as possible in the process, ultimately leading to improved cosmetic outcomes. Here we present the first ex-vivo results in patients that underwent BCS (*n* = 12) using an optical cross-polarized microcamera system. The main aim of this study is to assess the feasibility in detecting microcalcifications in the superficial layers of excised breast tissue.

## Methods

### Study population

Between March 2022 and June 2023, 12 patients were included in the Netherlands Cancer institute—Antoni van Leeuwenhoek hospital (NKI-AVL) that underwent BCS. Inclusion criteria were > 18 years and microcalcifications present in preoperative mammography. This study complied with the Declaration of Helsinki and was approved by the Institutional Review Board (IRB) of the NKI-AVL and registered with number IRBm20-086. Informed consent to participate in studies is acquired from all patients within the NKI-AVL.

### Cross-polarized microcamera setup

The microcamera setup and subsequent technical features are described in detail in our previous work^24^. In short, the setup consisted of a handheld probe containing four optical fibers and a 1.0 × 1.0 mm camera with cross-polarizers to attenuate surface reflections. Images with a field of view (FOV) of 1.0 × 1.0 cm and magnification up to 50 μm were generated of the region of interest. The optical fibers were connected to a multi-LED source containing five independent LEDs in the visible and near-infrared range: blue (450 nm), green (530 nm), red (660 nm) and near-infrared (850 and 940 nm). Each LED can be switched on and off independently, allowing for five multispectral images per measurement location. In addition, RGB images can be reconstructed by combining red, green and blue images. The most favorable wavelengths for microcalcification visualization were found to be in the near infra-red (NIR), however the visible wavelengths proved important for image guidance and co-location. The camera and LEDs were controlled with a custom-built user interface programmed in LabVIEW (LabVIEW National Instruments, Austin, Texas).

### Study protocol

Immediately after surgery, the resected specimen was brought to the department of Pathology. According to standard pathology protocol, resection margins were inked, for margin assessment. When frozen, the specimen was cross-sectioned in slices with a thickness between 2 and 5 mm and placed on an A4 plastic sheet. Next, the slices were brought to the department of Radiology where a slice mammography (X-Ray) was taken of all slices. After examination of this mammography, one or two slices with microcalcifications were selected for optical imaging. Each selected slice was placed in a macrocasette.

### Measurement protocol

**Fig. 1:**
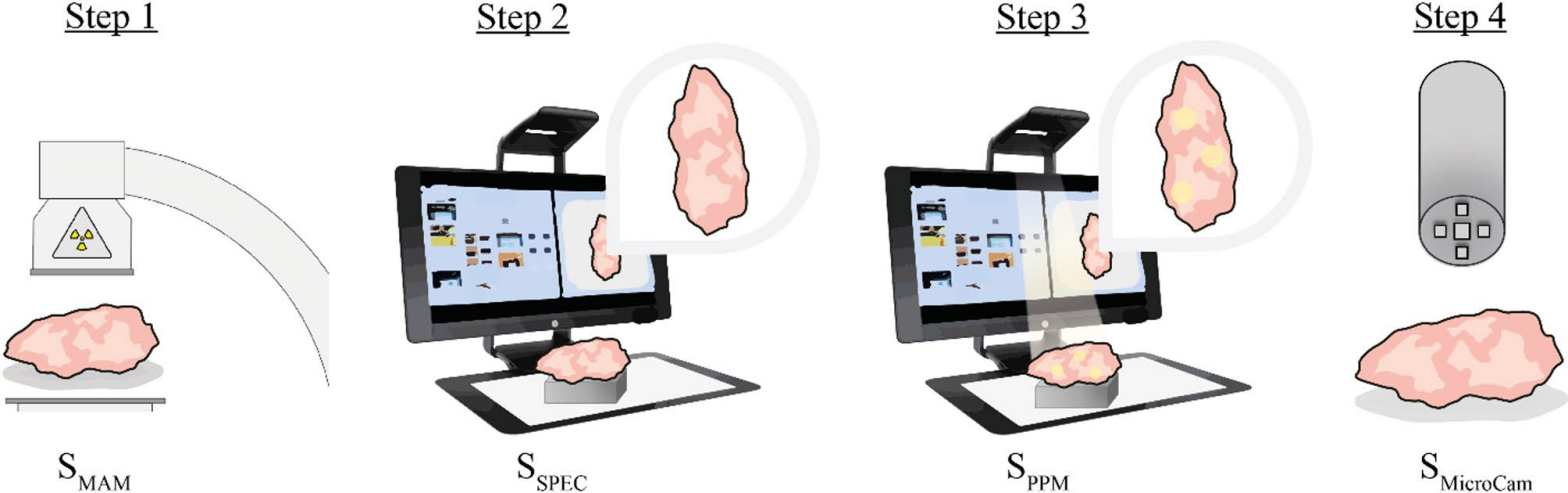
The steps of the measurement protocol are illustrated with the relevant image name indicated below the illustration. In Step 1 an X-ray of the slice is captured using a mammography system (S_MAM_). In Step 2 the PPM system^27^ is used to capture a RGB image of the slice (S_SPEC_). In Step 3 the PPM system is used to co-locate S_MAM_ with S_SPEC_ and dots are projected onto regions of interest to generate S_PPM_. In Step 4 the microcamera probe is placed above these regions of interest at a fixed distance of 8 mm from the tissue sample to achieve a field of view of 1.0 × 1.0 cm with upwards of 50 μm magnification. The multi-LED source provides the light used for illuminating the tissue. During acquisition, real-time images in color, greyscale and infrared are shown on the screen.

First, the measurement locations or regions of interest (ROIs), were identified in the mammography slice images (S_MAM_) based on the presence or absence (in the case of controls) of microcalcifications. The slice was placed under a Point Projection and Mapping (PPM) system^27^ which co-locates the x-ray image with an image of the slice. A top-down image from the breast tissue slice (S_SPEC_) was acquired from which 2–5 measurement locations were selected manually based on the ROIs. The selected measurement locations were then projected as light dots onto the specimen, and a new top-view image was acquired in order to track the measurement locations (S_PPM_). Measurement locations were selected in areas containing calcifications, and areas without. The handheld probe was placed at a fixed distance of 8 mm from the tissue for optimal focus. The center of the probe was carefully placed over the center of the projected measurement locations. Acquisitions were made by switching through the aforementioned LEDs of different wavelengths. In each measurement location, high-dynamic range (HDR) images were generated, resulting in 20–40 images for each wavelength per measurement location (S_MicroCam_). Afterwards, the slice(s) were returned to the department of Pathology and processed according to standard protocol. A schematic of the measurement protocol is shown in Fig. 1.

### Data visualization

**Fig. 2:**
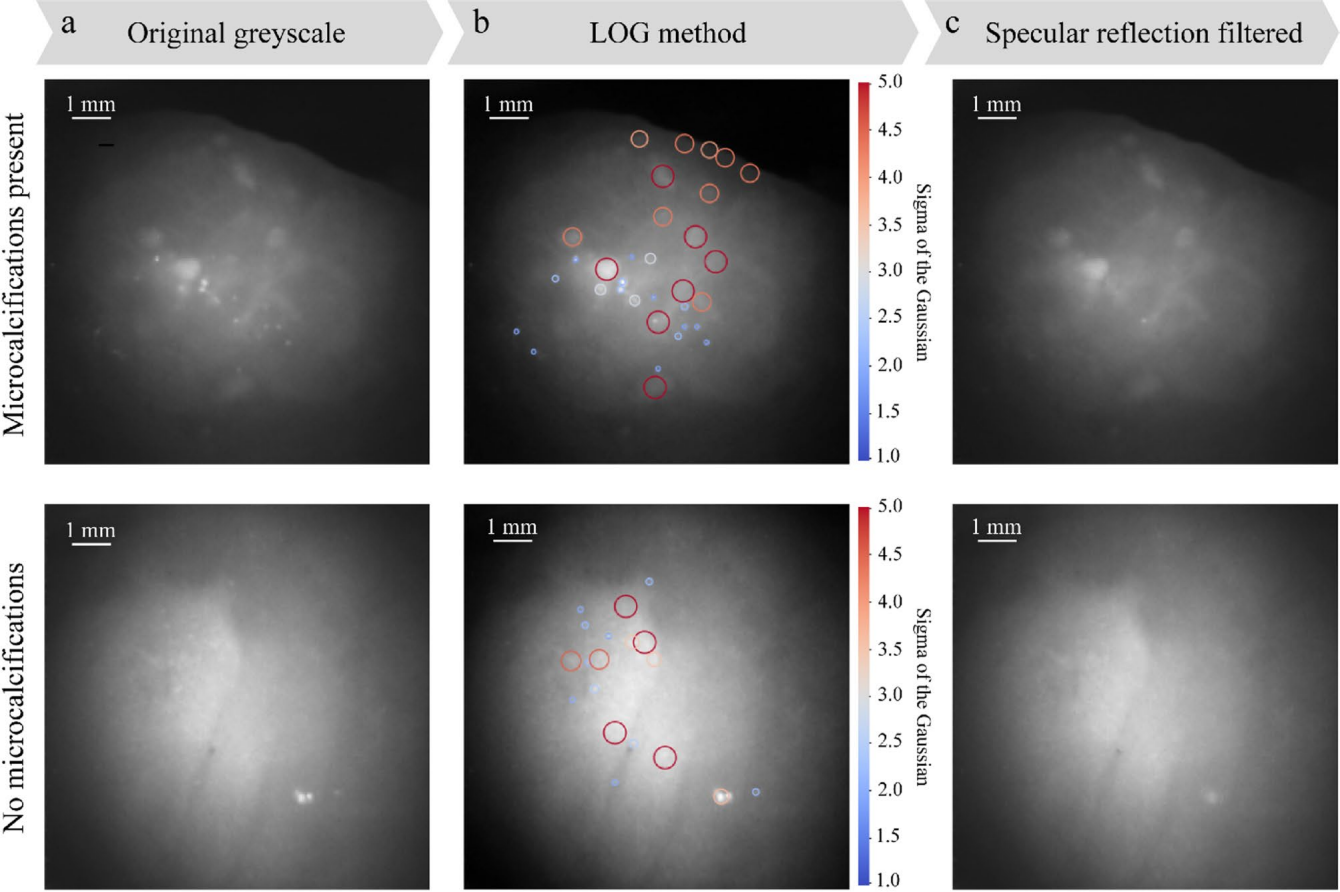
Examples of the method for filtering specular reflections from S_MicroCam_ images. (**a**) Original greyscale images with microcalcifications present and not present. (**b**) Blob-like features are detected in the images using a LOG method. The scale bar indicates the sigma of the gaussian filter used, which translates to the size of the blob-like feature detected. Larger detected blobs are circled in red, smaller ones in blue. (**c**) Specular reflection filtered images are generated. Blobs under a threshold size are filtered out using a median filtering technique.

The illumination fibers and camera are cross polarized to filter out specular surface reflections. However, under certain conditions some specular reflections remain due to the limited contrast ratio and angular performance of the polarizers. These reflections are present in the form of small bright peaks in the image. Using the Laplacian of Gaussian (LOG) method, blob-like features in the image can be identified. Such features include both specular reflections as well as other larger blob-like features in the image. The method is a multi-scale method, using a varying sigma of the Gaussian convolution to detect features of differing sizes. Subsequently, features below a threshold of 400 μm in size are excluded. This feature size is based on the size of microcalcifications, which can be as low as 300 μm, but mostly exceeds 400 µm^28^. In order to present a filtered image to the clinician, the locations of these excluded features can be median blurred to remove the sharp specular reflection peaks. The results of this process can be seen in Fig. 2. Figure 2a shows two raw greyscale images of different locations in S_SPEC_, with and without microcalcifications present, respectively. Figure 2b shows the output of the blob detection algorithm, with different colors indicating different sigma values and therefore different sizes of features. Figure 2c shows the corrected images. It is important to note that this visual correction can be beneficial when excluding features with certain criteria in a static measurement set up, however the impact of specular reflections is reduced when using the system to actively scan tissue. Specular reflections are easily identified in this case due to their movement with probe movement, whereas features in the tissue will remain in the image. Image visualization for a human observer can be further improved by employing a contrast limited adaptive histogram equalization (CLAHE) algorithm. CLAHE is a well-known computer vision technique which aims to increase contrast by redistributing pixel intensities across the full range of values while reducing the amplification of noise^29^.

### Data analysis

**Fig. 3:**
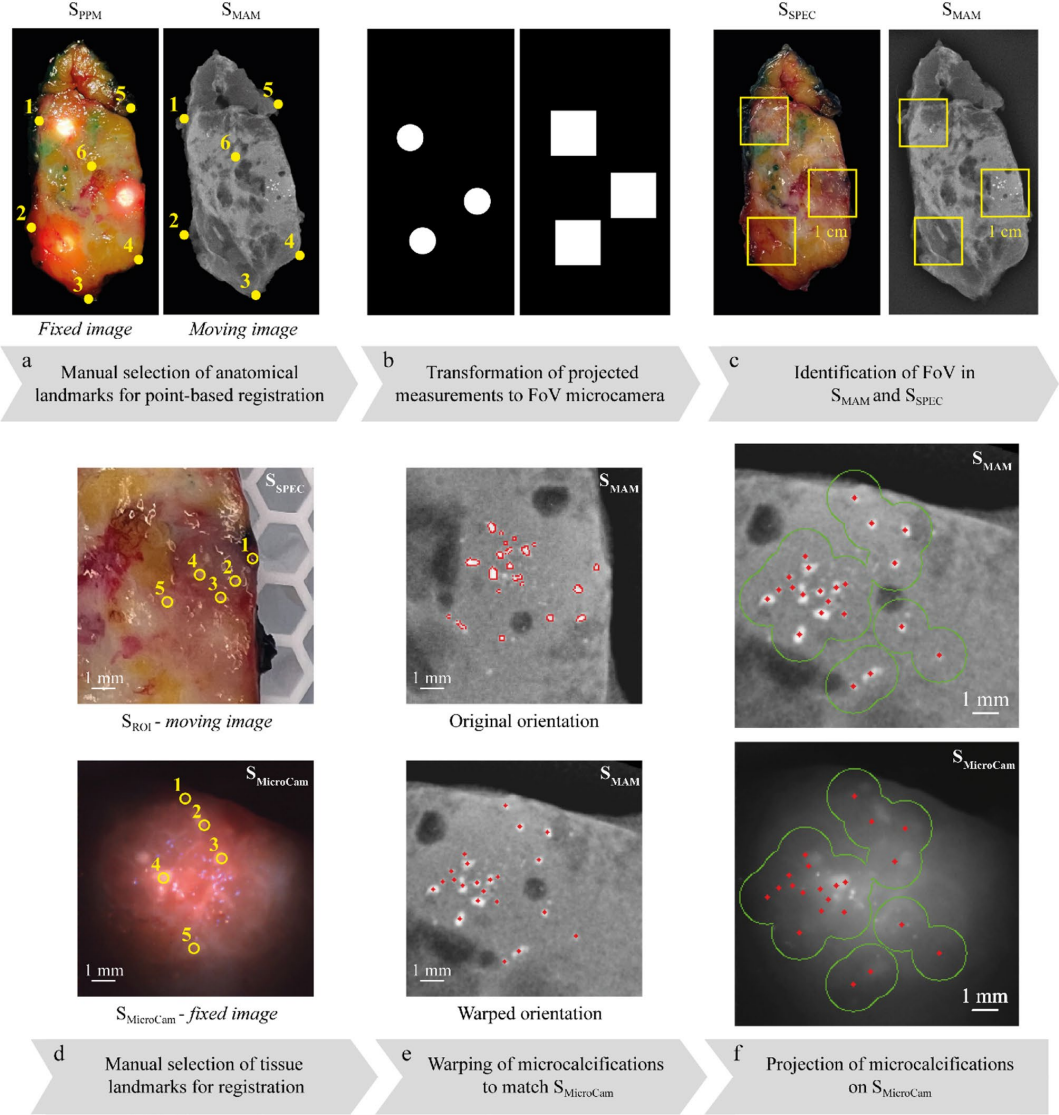
Workflow of the co-registration method for identification of microcalcifications in the S_MicroCam_ images. (**a**) The point-based image registration is performed by manually selecting corresponding anatomical landmarks in S_PPM_ and S_MAM_. (**b**) Binary image of the projected measurement locations were transformed into squares too match the FOV of the optical probe. (**c**) The measurement locations are overlaid on the S_PPM_ and S_MAM_, generating the exact counterpart of the S_MicroCam_ image. (**d**) S_ROI_ and S_MicroCam_ are co-located based on exactly the same visible surface landmarks on both images. In this step up to 20 anatomical landmarks are selected on each image. Six such points are shown as examples in the figure for readability. The landmarks were selected by a clinician who marked the locations using a custom script. (**e**) Based on the co-location in step 4, S_MAM_ is warped to match S_MicroCam_, thereby providing a new orientation of the microcalcifications. (**f**) Lastly, the microcalcifications from S_MAM_ are projected on S_MicroCam_ with a circle representing an error of 1 mm.

For identification of microcalcifications in the S_MicroCam_ images, multiple registration steps were performed (Fig. 3). A top-view image of the S_PPM_ was acquired as described in detail in a previous study from our institute^27^. Since there is limited deformation between S_MAM_ and S_PPM_ a point-based registration was performed, by selecting 6–8 corresponding anatomical landmarks in both images. Since the S_SPEC_ are squashed during the mammography, the S_PPM_ was chosen as the fixed image (Fig. 3a). Next, a binary image of the projected measurement locations was created by selecting the center point. The projected measurement points (circle with a true diameter of 0.5 cm) were transformed to match the FOV of the camera (square of 1.0 × 1.0 cm) (Fig. 3b). The binary FOV image was then overlaid with S_SPEC_ and S_MAM_. As a result, the exact measurement locations were identified in S_MAM_ (Fig. 3c).

The second registration involved the exact overlay of S_MicroCam_ and S_ROI_. For this, 15 and 20 corresponding anatomical landmarks were manually selected, after which S_ROI_ was transformed to fit S_MicroCam_ (Fig. 3d). Simultaneously, microcalcifications were identified and transformed to fit the orientation of S_MicroCam_ (Fig. 3e). Lastly, circles with a radius of 1 mm are drawn around the microcalcification in S_MAM_ and overlaid on the 850 nm S_MicroCam_ (Fig. 3f). Ultimately, four independent reviewers, two with medical knowledge and two with technical expertise, were asked to evaluate all S_MicroCam_ images. They had to score the images on the presence of microcalcifications, and performance scores were computed (sensitivity, specificity, accuracy and F-score).

### Statistical analysis

Statistical analysis was performed using IBM SPSS statistics v27 (SPSS Inc., United States) and Matlab (R2021b, the Mathworks Inc, Massachusetts). Qualitative data is presented as numbers and percentages.

## Results

12 patients, providing 22 tissue slices, were included in this study—baseline characteristics are shown in Table 1. In total 64 measurements were performed on locations with microcalcifications (35.9%) and without microcalcifications (64.1%). The majority of the patients underwent surgery as a result of DCIS (83.4%). The diameter of clustered microcalcifications was on average 30 mm and could be as large as 71 mm.

**Table 1:** Patient demographics.

	*N*	
Total patients	12	
Total WLE slices	22	
Measurement locations with microcalcifications	23	35.9%
Measurement locations without microcalcifications	41	64.1%
Gender		
Female	12	100%
Age, median (range)	55	52–62
Tumor type		
DCIS grade 1	1	8.3%
DCIS grade 2	2	16.6%
DCIS grade 3	7	58.3%
Lobular carcinoma in situ	1	8.3%
Invasive carcinoma	1	8.3%
Diameter of clustered microcalcifications, mm, mean (range)	30	5–71
Area of microcalcifications, mm^2^, mean (range)	22	1–143

### Ex-vivo study

**Fig. 4:**
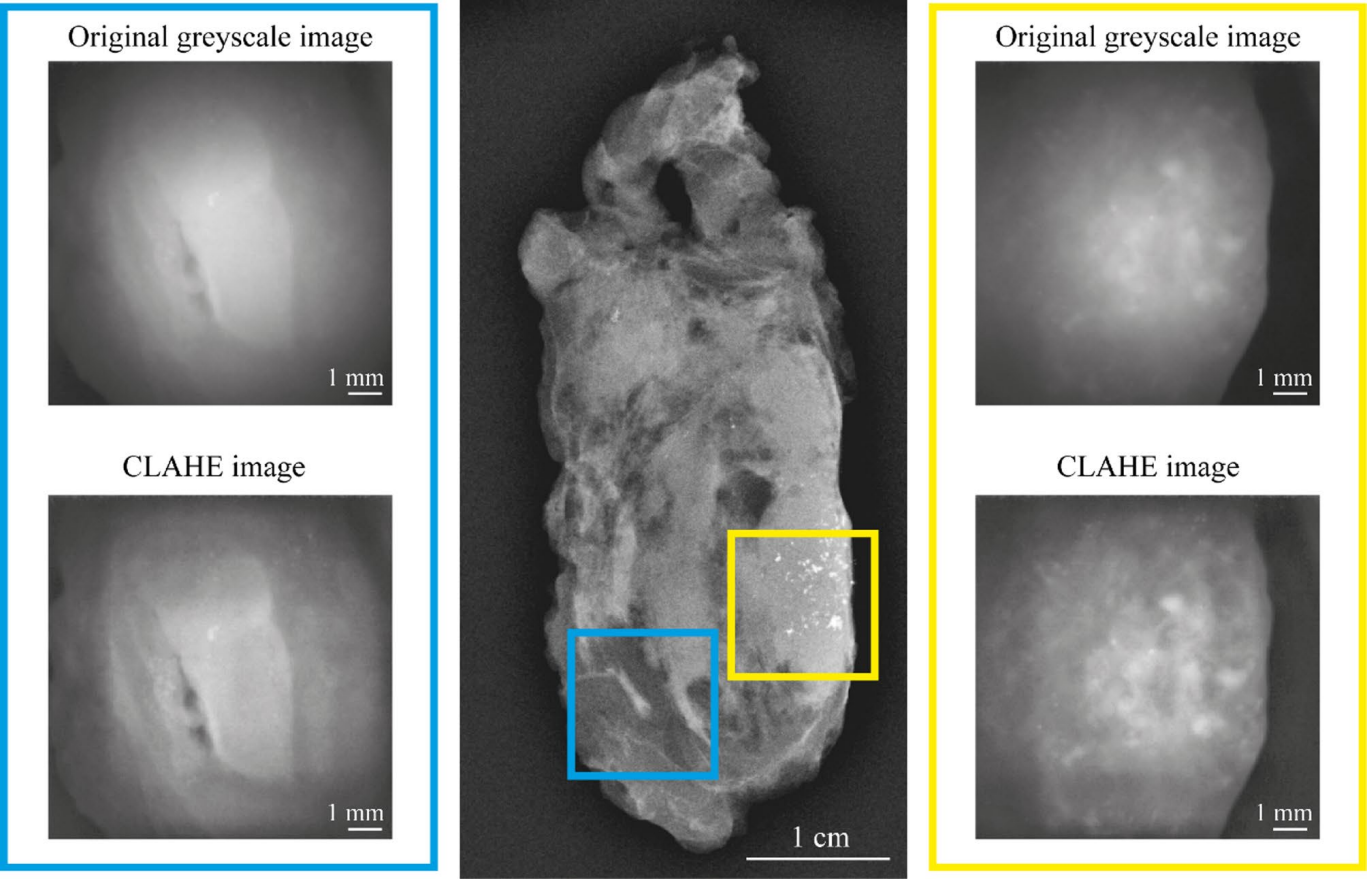
S_MAM_ with corresponding greyscale S_MicroCam_ images of near infrared light at 850 nm. The yellow box represents the measurement with microcalcifications, whereas the blue box is considered a negative control.

Each measurement location was assessed on the presence of microcalcifications in the S_MicroCam_ image and the corresponding S_MAM_ image, which was considered as ground truth. For every S_MicroCam_ image, CLAHE images were generated for improved feature differentiation. A typical example is shown in Fig. 4. When microcalcifications were present in the S_MicroCam_ images, they appeared highly reflective compared to the surrounding breast tissues (yellow box). When no microcalcifications were present, these features would be absent (blue box).

**Fig. 5:**
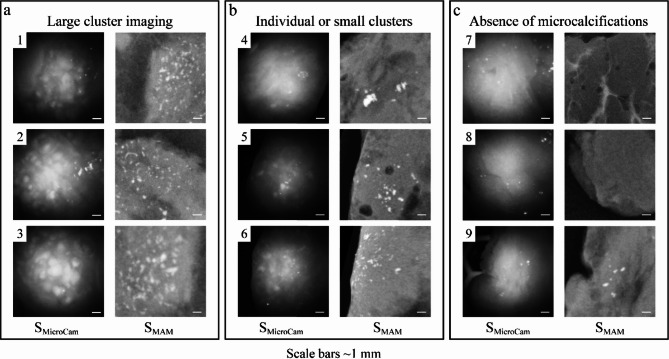
Nine examples of S_MicroCam_ images of various microcalcification distributions, accompanied with the corresponding S_MAM_. (**a**) Large clusters of microcalcifications within both S_MAM_ and S_MicroCam_. (**b**) Small groups of clustered microcalcifications, and (**c**) absence of microcalcifications in S_MicroCam_ images due to absence of microcalcifications in the mammography (examples 7 and 8), or suspected distribution at depth more than 2 mm (example 9).

The detectability of microcalcifications within the FOV of the microcamera was affected by the situated distribution and depth of the microcalcifications. Notably, microcalcifications situated within 2 mm of the tissue surface could be identified as bright features (Fig. 5a). However, in many cases microcalcifications were distributed throughout the depth of the complete tissue slice, leading to detection of only a fraction of the calcifications due to their depth below 2 mm (Fig. 5b). In negative control tissue slices, no features resembling microcalcifications were detected (Fig. 5c). Even so, microcalcifications that were situated below the imaging depth of 2 mm were not detected, while being observed in the control mammography (example 9). In Fig. 5c some residual specular reflections can be seen. These can be reduced with the aforementioned median filtering technique. Additionally when identifying calcifications larger diffuse fields of calcifications are of particular interest, and any specular reflections usually present as isolated bright spots. Individual bright spots are less clinically relevant. Further limitations are discussed in the discussion section.

Four independent reviewers were asked to evaluate all S_MicroCam_ images. They were tasked with the binary assessment of microcalcifications in the provided images, as shown in Table 2. Their collective analysis yielded an average sensitivity of 77.8% and specificity of 80.0%, resulting in an overall accuracy of 79.0%. The calculated average F-score stood at 73.8%.

**Table 2:** Independent reviewer assessment scores. The lower and upper confidence intervals for sensitivity, specificity, accuracy, and positive predictive value (PPV) at 95% confidence are reported in brackets.

	Sensitivity (%)	Specificity (%)	PPV (%)	Accuracy (%)	F-score (%)
Reviewer 1 (clinician)	73.9 (51.6, 89.8)	87.8 (73.8, 95.9)	77.3 (59.1, 88.9)	82.8 (71.3, 91.1)	75.6
Reviewer 2 (clinician)	79.2 (57.9, 92.9)	84.2 (68.8, 94.0)	76.0 (59.6, 87.2)	82.3 (70.5, 90.8)	77.6
Reviewer 3 (technician)	78.3 (56.3, 92.5)	75.6 (59.7, 87.6)	64.3 (50.2, 76.3)	76.6 (64.3, 86.3)	70.6
Reviewer 4 (technician)	80.0 (59.3.3, 93.2)	70.3 (53.0, 84.1)	64.5 (51.6, 75.6)	74.2 (61.5, 84.5)	71.4
Average	77.8	80.0	70.5	79.0	73.8

## Discussion

The present study demonstrates the first results of a cross-polarized microcamera imaging setup for detecting microcalcification in the superficial 2 mm of breast tissue. Measurements were performed on ex vivo breast specimens containing microcalcifications of varying sizes and distributions. Regions of interest of 1.0 cm^2^ were imaged, providing highly detailed features with a magnification up to 50 μm. These images were registered to the corresponding areas in the S_MAM_ with a registration method for identification of microcalcifications in S_MicroCam_ images (Fig. 3), developed in-house. The final images were independently scored by two medical specialists and two technical specialists, achieving an overall sensitivity of 77.8%, specificity of 80.0% and accuracy of 79.0%.

Up to 30% of all excised DCIS lesions result in tumor-positive resection margins after histopathology^6^, and currently there is no objective imaging technique available for real-time intraoperative assessment of the resection margin. Moreover, DCIS is well known for containing microcalcifications in 60–90% of cases, which are being formed and deposited at the invasive border of the lesion^4,7,8^, making them an interesting target for resection margin assessment. The golden standard for resection margin assessment remains histopathology, however this can take up to several days. Over the last years, new innovations in the operating theatres allow for scanning of the entire breast lump directly after excision^30^, having the potential of providing direct feedback to the surgeon. However, these techniques are not able to visualize the resection plane that remains in the breast after excision of the tumor.

One prominent advantage of the proposed microcamera setup is the possibility for real-time assessment of tissue structures during imaging and scanning large portions of tissue in a short amount of time. The current setup allows for fast switching between visualization of tissue structures on and below the tissue surface. Moreover, a microcamera setup presents a highly favorable approach for assessing the resection plane within the breast cavity post tumor excision. The compact and easily maneuverable nature of the microcamera probe, together with the real-time and high-resolution visualization capabilities enables meticulous evaluation of the cavity. This holds significance not only in visualizing calcifications but also in evaluating residual tumor burden. The small footprint of the camera allows for simple integration with other sensing modalities, and addition of a camera sensor on to existing surgical tools if desired.

The microcamera system utilizes cross-polarization, as described in detail in our recent work^26^. However, due to limitations in the contrast ratio of the polarizers not all specular reflections were removed. A trade-off was made between contrast ratio and transmission across a wide wavelength range when selecting polarizers for this work. Nonetheless, this is a technical challenge, and not a theoretical limit to the proposed microcamera technique. With high contrast polarizers at select wavelengths the removal of specular reflections could be improved further. Specular reflection reduction by cross-polarization is important when improving the overall image quality of such a small sensor, and when examining a small area of interest. It is important to note that these small remaining specular reflections are easily distinguished from features of interest, since they will change with different viewing positions and angles when moving the camera during a tissue scan. When showing static images, post processing can be employed to remove the remainder of the specular artifacts. In the present study, the remaining specular reflections were identified and eliminated during post-processing, improving the quality of S_MicroCam_ images and visualization of microcalcifications. Hence, from this study it can be concluded that specular reflections occurring at the tissue surface can obfuscate the microcalcifications that reside inside the tissue, limiting the performance metrics. By effectively eliminating the surface reflections, either with improved cross-polarizing filters or by post-processing, performance metrics will increase.

In the current study, an overall sensitivity of 77.8% was achieved in detecting microcalcifications in S_MicroCam_ images. At first glance, this sensitivity might appear relatively modest. However, in reality the thickness of the tissue slices (3–5 mm), and the fact that microcalcifications can be spread throughout the entire slice, might exceed the penetration depth of the used wavelengths (2 mm). When calcifications were present in the superficial 2 mm of the tissue slice, in almost all cases they were visible in the S_MicroCam_ images. However, when the calcifications were located deeper than 2 mm, they could not be seen, explaining the modest sensitivity. While this may sound as a limitation of the presented setup, the superficial 2 mm are considered clinically relevant for assessing resection margins after surgical resection of the tumor^31^. Hence, identification of calcifications in the superficial 2 mm would provide the clinician with information regarding the possibility of residual tumor cells in the resection margin. It is important to realize that this technique has only been validated on slice mammography with pre-identified microcalcifications. Thus, to be able to translate this technique to an in-vivo setting, further studies should be conducted, focusing lumpectomy specimens without X-ray support beforehand.

We strongly emphasise the importance of controlling slice thickness in a follow-on study to get a better measure of the true sensitivity of the apparatus. We also propose the use of a depth sensitive ground truth modality such as using a micro-CT system to investigate the slices and determine the depth of the micro-calcifications. In this way the depth of the calcification can be used to better understand the depth sensitivity of our system, and further validate the efficacy of using the technique in the context of 2 mm margins. We expect that both sensitivity and accuracy will consequently improve.

It is important to note that the described technique is only tested on the visualization of microcalcifications. However, due to the fact that DCIS is not accompanied by microcalcifications in 10–40% of cases, visualization of solely microcalcifications in the resection margins is likely insufficient to aid in decision making in the clinical setting. Therefore, the current approach should be tested in combination with a supplementary technique, such as DRS, or any other technique able to differentiate between breast tissue and tumor tissue with a high sensitivity. In recent studies, DRS was able to detect DCIS and IC with high accuracies, potentially aiding in surgical guidance intra and postoperative^16,32^. As such, the combination of DRS with the proposed microcamera setup in this study, might provide an accurate tool for resection margin assessment in BCS, by means of examining the excised tumor, as well as the tumor cavity for residual tumor/microcalcifications.

Due to the fact that suspicious microcalcifications are also located at the border of the tumor, they can provide an interesting target for intraoperative margin assessment^4^. Moreover, when microcalcifications are present in the resection plane after excision of the tumor, they are a strong indication that tumor cells are left behind in the patient. Therefore, visualizing microcalcifications in the resection plane during surgery can be pivotal in assessing resection margins intraoperatively, allowing surgeons to excise extra tissue in the same procedure when indicated.

## Conclusion

The presented novel cross-polarized microcamera setup provides a non-invasive technique for fast and real-time assessment of microcalcifications in superficial breast tissues. Through a robust registration method, accurate correlations were made between the microcamera images and corresponding slice mammography regions. By focusing on the context of the region of interest (superficial 2 mm), the current approach provides both promising results as well as considerable anticipation for future studies and applications.

### Data Availability

The datasets used and/or analyzed during the current study are available from the corresponding author on reasonable request.

## Data Availability

The datasets used and/or analyzed during the current study are available from the corresponding author on reasonable request.
